# A systemic increase in the recombination frequency upon local infection of *Arabidopsis thaliana* plants with oilseed rape mosaic virus depends on plant age, the initial inoculum concentration and the time for virus replication

**DOI:** 10.3389/fpls.2013.00061

**Published:** 2013-03-21

**Authors:** Youli Yao, Palak Kathiria, Igor Kovalchuk

**Affiliations:** Department of Biological Sciences, University of LethbridgeLethbridge, AB, Canada

**Keywords:** *Arabidopsis thaliana*, oilseed rape mosaic virus, homologous recombination frequency, genome stability

## Abstract

In the past, we showed that local infection of tobacco leaves with either tobacco mosaic virus or oilseed rape mosaic virus (ORMV) resulted in a systemic increase in the homologous recombination frequency (HRF). Later on, we showed that a similar phenomenon occurs in *Arabidopsis thaliana* plants infected with ORMV. Here, we tested whether the time of removing the infected leaves as well as viral titer have any effect on the degree of changes in HRF in systemic tissues. An increase in HRF in systemic non-infected tissues was more pronounced when the infected leaves were detached from the infected plants at 60–96 h post-infection, rather than at earlier time. Next, we found that exposure to higher concentrations of inoculum was much more efficient in triggering an increase in HRF than exposure to lower concentrations. Finally, we showed that older plants exhibited a higher increase in HRF than younger plants. We found that an increase in genome instability in systemic tissues of locally infected plants depends on plant age, the concentration of initial inoculums and the time of viral replication.

## INTRODUCTION

Viral infection is a common stress for plants. Typically, plants respond to such attack by activating various defense pathways. The efficiency and speed of plant response, however, depend on the presence of genes of resistance (*R*-genes) that allow plants to recognize pathogen avirulence genes (*Avr*-genes; Durrant and Dong, 2004). The recognition of a pathogen through gene-for-gene interactions results in an incompatible response that manifests itself in the salicylic acid (SA)-dependent restriction of virus movement and the activation of systemic defense against future pathogen attacks known as systemic acquired resistance (Durrant and Dong, 2004; Scholthof, 2004). In contrast, if either the *R*-gene or the *Avr*-gene is missing, interactions between pathogens and plants are compatible; viruses are able to spread and infect plants systemically (Dong, 2001; Scholthof, 2004).

Previously, we showed that a compatible interaction between SR1 tobacco plants and either tobacco mosaic virus (TMV) or oilseed rape mosaic virus (ORMV) results in an increase in the frequency of homologous recombination (HRF; Kovalchuk et al., 2003). We demonstrated that when infected leaves were removed from plants 24 h after infection, a virus was not able to spread to systemic tissues, but an increase in HRF was observed throughout the plant. Curiously, incompatible interactions between TMV/ORMV and tobacco cultivar Big Havana plants that have the gene of resistance against these viruses did not result in an increase in HRF (Kovalchuk et al., 2003). We hypothesized that a compatible interaction triggers the production of a systemic signal that spreads faster than a virus and promotes an increase in HRF in non-infected tissues.

It was not clear whether a similar phenomenon of a systemic increase in HRF in response to local infection occurs in other plants, therefore, we recently tested this phenomenon in *Arabidopsis thaliana*. Infection of *Arabidopsis* plants with TMV was documented before (Padmanabhan et al., 2005), however, it is believed that infection with ORMV is more efficient (Hu et al., 2011). *Arabidopsis* cultivars differ in their sensitivity to ORMV, though infection of Col-0 with ORMV is very efficient (Martín Martín et al., 1997). Fourteen different *Arabidopsis* cultivars infected with TMV exhibited that Col-0 plants were poorly infected (Dardick et al., 2000). Although we were not able to find a publication describing direct comparisons of the efficiency of infection between TMV and ORMV, a similar comparison between *Arabidopsis* plants infected with cucumber mosaic virus strain Y (CMV-Y) and ORMV showed that ORMV was more efficient (Huang et al., 2005).

We tested the influence of local infection of *Arabidopsis* with ORMV and found that it also resulted in a systemic increase in HRF as well as in an increase in the frequency of point mutations and microsatellite instability (Yao et al., 2011). Curiously, changes in microsatellite stability and mutation frequency were more pronounced than changes in HRF. Similar results were seen in the progeny of infected tobacco plants where rearrangements in resistance gene loci occurred at a higher frequency than in housekeeping genes (Boyko et al., 2007). The rearrangements observed may have been the result of point mutations, deletions or recombination events.

The following questions were addressed in this manuscript. Does the plant age influence changes in HRF in systemic tissues of infected plants? Does the concentration of initial inoculum influence the degree of an increase in HRF? Does the HRF increase depend on the time of virus replication in infected leaves? Here, we demonstrate that an increase in HRF positively correlates with plant age, the concentration of initial inoculums and the time for virus replication in locally infected leaves.

## RESULTS

**FIGURE 1:**
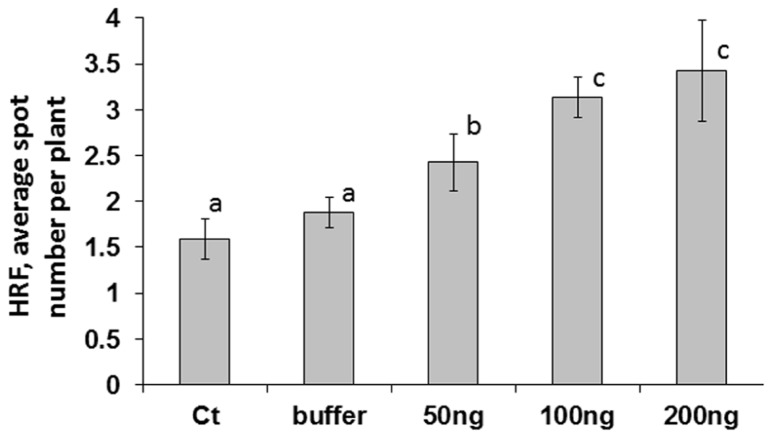
**An increase in HRF in systemic tissues of locally infected *Arabidopsis* plants depends on virus amount used for infection**. Plants from line 15d8 were infected at 3 weeks of age, infected leaves were removed at 48 hpi, and HRF was analyzed one week later. “Ct” – non-infected plants. “Buffer” – buffer-treated plants. “50 ng”, “100 ng”, “200 ng” – plants infected with 50, 100, or 200 ng ORMV, respectively. The data are shown as the average number of recombination events per plant with SE calculated from three independent repetitions (20 plants in each experimental group). The letters indicate significantly different values (*P *< 0.05); similar letters indicate statistically similar values.

A higher viral titer is typically associated with faster systemic spread of the virus; the *Tomato yellow leaf curl virus* titer correlated positively with the amount of initial inoculums used for infecting tomato plants (Kunik et al., 1994). We hypothesized that the concentration of initial inoculums would have different results on an increase in HRF. While analyzing HRF in plants infected with 50, 100, or 200 ng of ORMV (the infected leaves were removed at 48 hpi (hours post-infection), we found that HRF was the highest in plants infected with 200 ng and the lowest in those ones infected with 50 ng ORMV (**Figure 1**). There was a strong positive correlation between the amount of virus used to initiate the infection and an increase in HRF (*r* = 0.726). The differences in HRF between plants infected with 100 and 200 ng were not significant so (*P *> 0.1).

Since the previous experiment showed that the highest concentration of primary inoculums resulted in the highest increase in HRF, we hypothesized that an increase in HRF is more or less proportional to the amount of viral RNA initially encountered by plants. It was not clear whether viral replication was a determinant of the process. We hypothesized that if the virus is allowed to propagate in infected leaves for a longer period of time, we might observe a stronger induction of HRF. Since it was not clear whether the presence of virus in the systemic leaves used for the analysis of HRF would influence the results, we had to determine the time of systemic spread of the virus from the initial site of infection.

**FIGURE 2:**
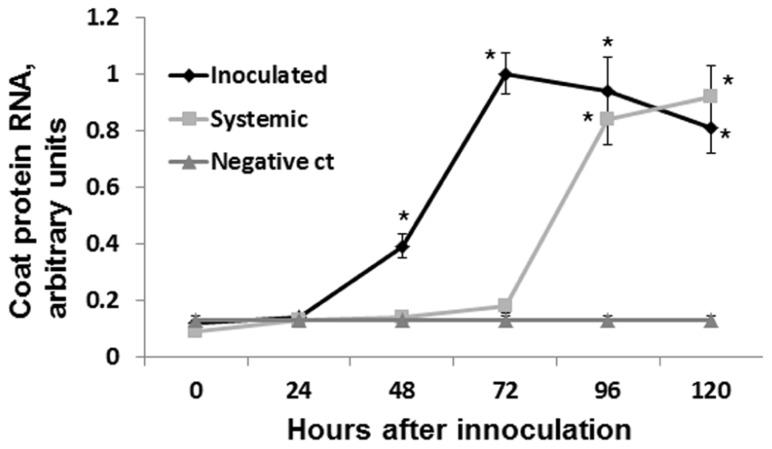
**Detecting coat protein mRNA**. Plants were infected with 100 ng of ORMV, both infected and non-infected leaves were taken for the analysis at different time points (from 0 to 5 days). Four independent plants were used per each experimental time point. mRNA was isolated, converted to cDNA and used to amplify coat protein RNA via qRT-PCR. ORMV was converted to cDNA and used as a negative control in the amplification (no amplification was found in this sample). Negative control sample contained no gDNA/cDNA. *Y* axis shows arbitrary units of cDNA amplified using primers against gene coding for coat protein. *X* axis shows time in hours after infection. Data are shown in arbitrary units (with SD). Asterisks show significant difference from negative control (*P *< 0.05).

To test the speed of virus spread to the systemic leaves, we infected several groups of plants with ORMV (100 ng of per plant) and cut leaves at 24, 48, 72, and 96 hpi. We analyzed the presence of viral RNA in the systemic leaves by performing the quantitative real-time PCR (qRT-PCR). We found the presence of viral RNA in the sample from the 96 hpi group but not in the samples from the 72, 48, or 24 hpi groups (**Figure 2**). Therefore, we concluded that the virus spreads systemically between 72 and 96 hpi.

**FIGURE 3:**
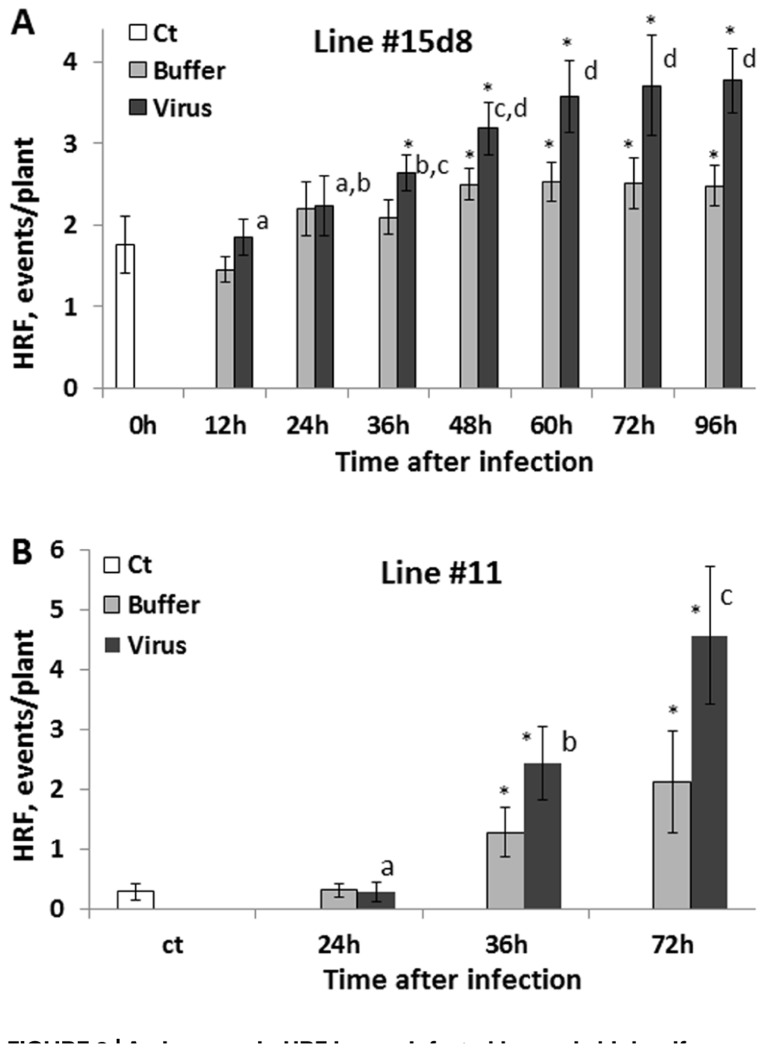
**An increase in HRF in non-infected leaves is higher if infected leaves are removed later after infection**. Plants from line 15d8 **(A)** and line #11**(B)** were infected with 100 ng ORMV (Virus), treated with buffer only (Buffer) or not treated at all (Ct). Infected leaves were removed at different time points from 12 to 96 hpi. HRF was analyzed in non-infected tissues 1 week after infection. The data are shown as the average HRF per plant (calculated from three repeats, each consisting of 20 plants, with SE). The asterisks show significant differences between the virus-and buffer-treated groups or buffer-treated and control plants (*P *< 0.05). The letters indicate whether there is a significant difference between time points in the virus-treated groups.

Next, we analyzed HRF in systemic tissues of plants infected with 100 ng virus when the infected leaves were removed at 12, 24, 36, 48, 60, 72, and 96 hpi. We found that an increase in HRF was the highest in the 60, 72, and 96 hpi groups, with the results being statistically similar among these three groups (*P *> 0.1; **Figure 3A**). Remarkably, HRF did not significantly increase in the 12 and 24 hpi groups as compared to the control one (*P *> 0.1). To confirm these data, we performed a similar experiment with a different transgenic line, line 11 (C24 cultivar) carrying in the genome the β-glucuronidase-based recombination substrate. For this line, we used only three time points: 24, 36, and 72 hpi (**Figure 3B**). A statistically significant increase in HRF was observed at 36 hpi (*P *< 0.05) and 72 hpi (*P *< 0.01) but not at 24 hpi (*P *< 0.1) as compared to the control. A significant increase in the HRF was also observed in several buffer-treated groups, especially in those in which buffer-treated leaves were removed at 72–96 h post-treatment. This experiment has confirmed that an increase in HRF in *Arabidopsis* plants occurs only when the virus is allowed to propagate in infected leaves for more than 24 h and showed that wounding stress potentiates the HRF increase.

**FIGURE 4:**
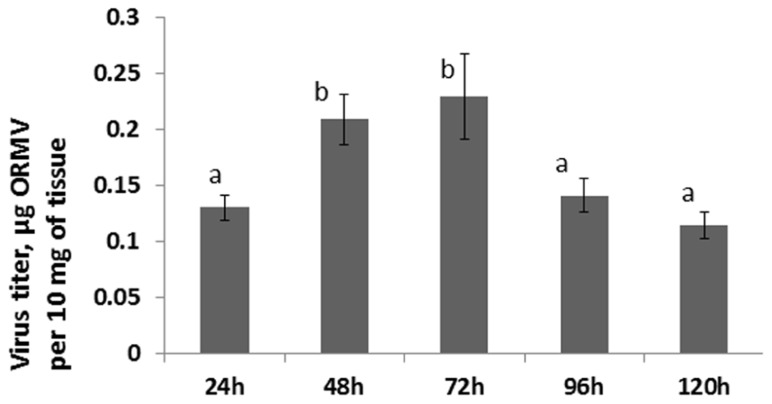
**Viral titer in infected leaves**. Plants from line 15d8 were infected with 100 ng ORMV, and a viral titer was measured in infected leaves at different time points from 24 to 120 hpi. The data are shown in microgram of ORMV per 10 mg of plant tissue (calculated from four plants per each of three repetitions, with SE). The letters indicate whether there is a significant difference between time points in the virus-treated groups.

Since no statistically significant increase was observed in the12 and 24 hpi groups, we hypothesized that an increase in HRF in systemic tissues requires certain viral replication in infected tissues. The analysis of viral concentration in the infected leaves showed a significant (*P *< 0.01) increase between 24 and 48 hpi (**Figure 4**). Viral titer picked at 72 hpi and the analysis at 96 and 120 hpi showed a significant decrease (*P *< 0.01 for both) as compared to that at 72 hpi. We hypothesized that a decrease in the viral titer in the infected leaves observed at 96 hpi could be due to systemic spread of the virus. Indeed, the analysis of ORMV RNA in non-infected tissues showed a substantial accumulation of virus at 96 hpi which increased at 120 hpi (**Figure 2**).

It was surprising that an increase in HRF was not much greater in the 96 hpi group as compared to the 72 hpi group (**Figure 3A**). Initially, we thought that the presence of the virus in the tissues would lead to a further increase in recombination, but as it has been shown above, it is not the case. It is possible that the system of recombination response saturates after plants are exposed to a certain amount of inoculums. Indeed, the influence of infection with 200 ng of virus was not different compared to 100 ng. Similarly, the 72 hpi group did not substantially differ from either the 60 hpi group or the 96 hpi group for that matter.

**FIGURE 5:**
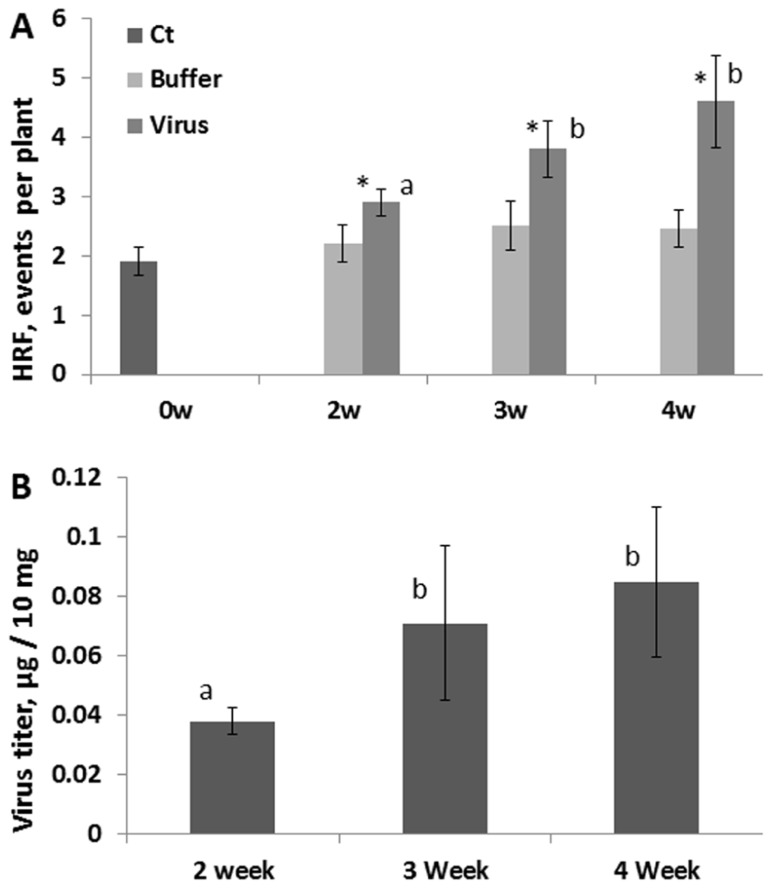
**HRF and viral titer in plants infected at different time points during development**. Plants from line 15d8 were infected with 100 ng ORMV at 2, 3, and 4 weeks after germination. **(A)** HRF was analyzed in non-infected tissues 1 week after infection. The data are shown as the average HRF per plant (calculated from three repeats, each consisting of 20 plants, with SE). “0w” shows the HRF for control (Ct) – non-infected plants. “Buffer” and “Virus” show buffer-treated and virus-infected plants, respectively. The asterisks show significant differences between the virus- and buffer-treated groups (*P *< 0.05). The letters indicate whether there is a significant difference between time points in the virus-treated groups. **(B)** A viral titer was measured in infected leaves at 48 hpi. The data are shown in microgram of ORMV per 10 mg of plant tissue (with SE). Four plants per each data point were analyzed. The letters indicate whether there is a significant difference between time points in the virus-treated groups.

We next analyzed whether the HRF increase would also depend on whether plants were infected at younger or older age. We infected 2-, 3- and 4-week-old plants with 100 ng of virus and removed the infected leaves at 48 hpi. While analyzing HRF in systemic tissues 1 week post-infection, we found that an increase in HRF was the highest in the oldest plants (**Figure 5A**). The analysis of viral titers in the removed infected leaves (48 hpi) showed the highest accumulation of virus in the oldest leaves (**Figure 5B**). This suggests that viral infection progresses faster in older leaves allowing for higher concentrations of virus. This, in turn, results in a higher increase in HRF in older plants.

**FIGURE 6:**
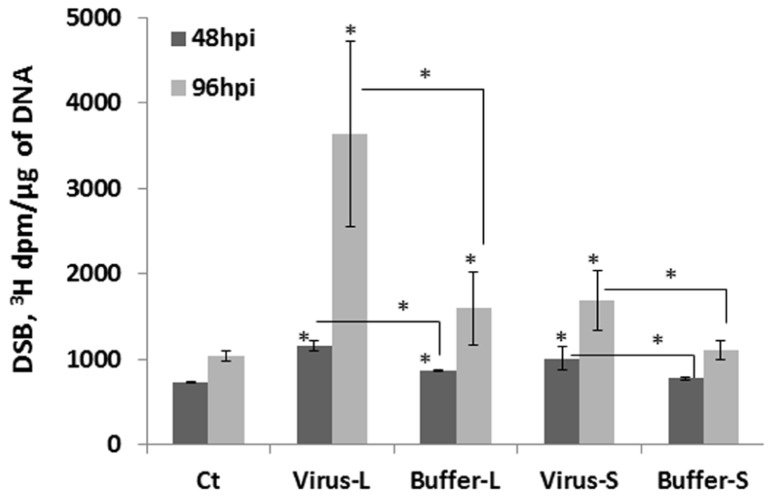
**The level of double-strand breaks in infected and non-infected leaves**. Plants from line 15d8 were either infected with 100 ng ORMV or treated with buffer. The treated and non-treated leaves from four different plants per treatment were taken for the analysis of double strand breaks (DSBs) by the ROPS assay. The data are shown as ^3^H incorporation in dpm/μg of DNA. The asterisks show a significant difference between the treated groups (virus or buffer) and the control as well as between the virus-treated and buffer-treated groups (*P *< 0.05).

What could be the reason for an increase in HRF in unexposed tissues? It is possible that viral infection results in an increase in the number of strand breaks, perhaps through local changes in chromatin structure or/and changes in the expression of repair genes involved in homologous recombination. To test whether the levels of strand breaks change in infected and non-infected tissues, we analyzed the presence of strand breaks using the random oligonucleotide-primed synthesis (ROPS) assay. We found that the levels of strand breaks increased in infected and non-infected leaves at 48 and 96 hpi, with changes in the 96 hpi group being more dramatic (**Figure 6**). The levels of strand breaks also increased in the buffer control groups, presumably as a result of a response to mechanical wounding when plants were treated with buffer and carborundum. The level of strand breaks was always higher in the virus-treated groups as compared to the buffer-treated ones (*P *< 0.05 in all cases). When infected leaves were removed from plants at 48 hpi, a further accumulation of strand breaks was observed in non-infected leaves tested at 96 hpi, and the level of strand breaks was much higher than that in non-infected leaves at 48 hpi (data not shown). This suggests that there is an active process running in non-infected leaves that result in a further increase in the level of strand breaks.

## DISCUSSION

Our experiments show detectible ORMV presence in systemic tissue 4 days after inoculation. Unfortunately, we could not find any published literature on the speed of systemic movement of ORMV in *Arabidopsis* that could confirm our findings. One report quoting unpublished data suggests that ORMV moves systemically in *Arabidopsis* in about 3 days, although the symptoms do not appear until day 10 post-infection (Hu et al., 2011). It is not clear what techniques have been used by these authors to detect the presence of virus, but it is possible that the methods were more sensitive than ours. We can only hypothesize that the authors were able to detect some virus-derived small interfering RNAs (Hu et al., 2011). It is not clear whether such molecules are indeed present in systemic tissues at 3 days post-infection and whether they are able to cause changes in HRF, but such hypothesis would be interesting to test. Although we have not detected viral RNA at 3 days post-infection, we can exclude that our real time RT-PCR was not sensitive enough to detect viral presence and thus we cannot exclude some effect of this viral RNA on HRF in the systemic tissues. Alternatively, it is possible that other viral components, such as movement protein (MP), could have reached the systemic tissue and trigger the response leading to HRF increase.

Analysis of the viral titer in the infected tissue showed initial accumulation picking at 72 hpi, followed by a drop in a viral titer at further time points. Although the data on the accumulation of ORMV in *Arabidopsis* is not available, similar measurements in TMV-infected tobacco plants showed that the viral titer reached the maximum at 48 hpi and then gradually decreased reaching the minimum value at 120 hpi (Deom et al., 1990). Similarly, infection of tomato plants with TMV resulted in a gradual increase in viral concentration observed between day 1 and day 7 followed by a steady decline at later days (Balogun et al., 2002).

The data on the correlation between the efficiency of viral progression and plant age are controversial; and it depends on the type of viruses, plants as well as the type of tissues used for infection. The end-point that has been measured, such a viral titer or the appearance of symptoms also plays a role. The analysis of the accumulation of MP of TMV in the infected tobacco plants showed much higher amounts of TMV in older leaves as compared in younger leaves (Deom et al., 1990). Moreover, injection of liposomes containing MPs into leaves of different age also showed a more efficient movement in older leaves vs younger leaves (Deom et al., 1990). On the other hand, in potato, susceptibility to potato leafroll virus was shown to decrease with plant age (DiFonzo et al., 1994). Mature pepper plants were also shown to substantially resist tomato spotted wilt Tospovirus (Beaudoin et al., 2009). The effect of tomato yellow leaf curl virus on tomato yield was also age-dependent; the lowest yield was observed in plants infected earlier during development (Levy and Lapidot, 2008).

It is still unclear what could be the primary cause of the HRF increase in systemic tissue of infected plants. Moreover, we are not absolutely sure what is the mechanism of induction of HRF in the infected tissue, although it can be argued that a direct damage by free radicals could be one of the reasons. Indeed, in past we were able to show that pre-treatment with radical-scavenging compounds can partially alleviate such increase in both locally stressed and systemic tissue (Filkowski et al., 2004). It is possible that increase in HRF in systemic tissue is one of the responses to increased levels of free radicals. Although we have not detected the presence of ORMV in the systemic tissue at 3 dpi, it is possible that viRNAs do accumulate in systemic tissue at this time. If viral small RNAs accumulate in non-infected tissues, they may trigger changes in chromatin structure by binding DNA during replication/transcription or may cause genome instability directly. It is also possible that viral small RNAs may indirectly dysregulate either DNA repair or the chromatin modification machinery by targeting mRNA of responsible genes. One of the recent reports suggest that small non-coding RNAs are indeed able to regulate DNA repair – a novel class for small RNAs – double-strand break-induced small RNAs (diRNAs) have been recently identified (Wei et al., 2012). It is not impossible that viRNAs may have similar capacity to regulate strand break repair as diRNAs. It would be interesting in the future to inject a set of several artificial viRNAs and observe changes in HRF.

Several previous publications suggest that local exposure of plants to stress results in genetic instability in systemic tissues (Lucht et al., 2002; Kovalchuk et al., 2003; Filkowski et al., 2004; Yao et al., 2011). Stresses like ionizing radiation, UV-C, paraquat, infection with ORMV or *Peronospora parasitica* had an impact on somatic HRF, whereas exposure to TMV was shown to cause an increase in meiotic HRF. Furthermore, exposure to stresses like UV-B/UV-C, temperature fluctuations, water availability, exposure to salt, paraquat, and heavy metals as well as infection with TMV was showed to lead to an increase in somatic HRF in the progeny of exposed plants (Molinier et al., 2006; Pecinka et al., 2009; Boyko and Kovalchuk, 2010; Boyko et al., 2010; Kathiria et al., 2010). An increase in the recombination frequency in the virus-free progeny of tomato plants infected with TMV and potato virus X was also observed in an independent experimental system that did not use transgenic plants that allowed the detection of recombination events (Marii and Chiriac, 2009).

The paper by Lucht et al. (2002) showed that exposure to the fungal pathogen *P. parasitica* resulted in an increase in somatic HRF (Lucht et al., 2002). Similarly, the authors showed that exposure to BTH, a chemical replacement for SA, also resulted in an increase in HRF. Since SA is a key molecule that is activated in response to many pathogens, one could suggest that the pathways involved in response to pathogens probably cross-interact with either the DNA repair pathway or the pathway regulating chromatin structure. Indeed, one of the recent papers by Xin Dong’s group sheds a bit more light on the link between pathogen infection and homologous recombination (Durrant et al., 2007). SA is an inducer of the expression of pathogenesis-related (PR) genes. Transcription of PR genes in *Arabidopsis* depends on the function of the co-activator NPR1 and the repressor SNI1. The authors showed that SNI1 and RAD51D, proteins involved in homologous recombination repair, regulate gene expression, and DNA recombination. It was found that the NPR1-independent expression of PR genes requires an active RAD51D protein. Moreover, the *rad51d* mutant that is impaired in homologous recombination also has an enhanced disease susceptibility. The authors hypothesize that a dual role of RAD51D and SNI1 in the regulation of PR genes and homologous recombination suggests a link between a short-term defense response against pathogens and a long-term survival strategy (Durrant et al., 2007). Further connections between pathogen infections, pathogen responses and the recombination machinery were provided by Wang et al. (2010). The authors demonstrated that RAD51, one of the key proteins involved in the recombination process, is recruited to the promoters of several defense genes when a SA-dependent mechanism of systemic acquired resistance (SAR) is activated (Wang et al., 2010). It can be hypothesized that SAR or SAR-like events occurring in the systemic tissue of non-infected *Arabidopsis* plants activate the homologous recombination machinery leading to an increase in HRF. It would also explain why the higher inoculum concentration and prolonged viral replication (when infected leaves are cut off at later time points) have a stronger effect on an increase in HRF.

To summarize, our results demonstrated that the extent of systemic increases in HRF in plants that were locally infected with ORMV depends on many parameters, including the concentration of initial inoculums and the time of viral replication. We believe that the degree of increases in HRF primarily depends on viral concentration and the speed of viral replication. It remains to be established whether pathogen infection of *Arabidopsis* results in transgenerational changes as it was observed in tobacco plants (Kathiria et al., 2010).

## MATERIALS AND METHODS

### PLANTS USED

For the experiments, we used two transgenic *A. thaliana* lines, line 11 (C24) that carries in the genome a single copy of the β-glucuronidase (GUS)-based recombination substrate (Swoboda et al., 1994) and line 15d8 (Col-0) that carries in the genome a single copy of the luciferase (LUC)-based recombination substrate (Ilnytskyy et al., 2004). Recombination substrate consists of two non-functional truncated overlapping copies of a transgene (GUS or LUC). Strand break in one of the regions of homology can potentially be repaired by HR, resulting in restoration of transgene structure, thus allowing its detection *in vitro* (GUS) or *in vivo* (LUC).

### OILSEED RAPE MOSAIC VIRUS INFECTION

*Arabidopsis thaliana* plants were germinated and grown in soil under 16 h day/8 h night at 22°C. Two leaves of *A. thaliana* plants were inoculated with different amounts of ORMV at the age of 3 weeks as previously described (Boyko et al., 2007), the total amount of 50, 100 or 200 ng of the virus per plant. Mock-treated plants were inoculated only with phosphate buffer and served as wounding controls. To test changes in HRF as a function of age, plants were infected at 2, 3 and 4 weeks of age using 100 ng of the virus per plant. In both cases, leaves were removed at 48 hpi. Each experimental group consisted of 16–20 plants and each experiment was triplicated.

### HISTOCHEMICAL STAINING PROCEDURE

Histochemical staining, as described by Jefferson et al. (1987), was performed 1 week after infection (Jefferson et al., 1987). For destructive staining, plants were vacuum-infiltrated for 2 × 10 min in sterile staining buffer containing 100 mg of a 5-bromo-4-chloro-3-indolyl glucuronide (X-glu) substrate (Jersey Labs Inc., USA) in 300 ml 100 mM phosphate buffer (pH 7.0), 0.05% NaN3, 0.1% Triton X-100. Afterward, plants were incubated at 37°C during 48 h and bleached with ethanol.

### MEASUREMENT OF DOUBLE STRAND BREAK LEVELS

Quantification of 3′OH DNA breaks was performed using the ROPS assay (Yao et al., 2011). Infected or non-infected leaves harvested from four plants per each experimental group were used. The experiment was performed three times and average and SD were calculated.

### VISUALIZATION OF LUCIFERASE ACTIVITY

Recombination events in transgenic plants were visualized in living plants with a CCD camera 1 week after infection, as previously described (Boyko et al., 2006). HRF was calculated by counting the number of individual recombination events with CCD camera software and relating these numbers to the total number of plants in individual experimental group. Each experimental group consisted of ~20 plants, and each experiment was repeated three times.

### ANALYSIS OF VIRAL RNA

The analysis of the RNA level of the ORMV coat protein was done as described before (Yao et al., 2012). RNA was isolated from the leaves taken from four plants per experimental group. In brief, qRT-PCR) reactions were setup in the SsoFast EvaGreen Supermix (Bio-Rad), and the results were processed by Bio-Rad CFX manager software (v2.0), with tubulin as a reference. Oligo specificity of was checked by the melting curve analysis performed by the Bio-Rad CFX96 PCR machine after 45 amplification cycles and by gel-electrophoretic analysis. The coat protein RNA was amplified with: 5′-CTCGAATCAGTACCAGTATT-3′ and 5′-CTTCAGTTTCAATGATCCTA-3′ primers. Tubulin was amplified using 5′-ACAGAAGCGGAGAGCAACAT-3′ and 5′-TCCTCATCCTCGTAGTCACCTT-3′ primers. Two technical repeats were done pear each of three independent experiments.

### ANALYSIS OF THE VIRAL TITER

Viral RNA was extracted, and virus concentration was analyzed as previously describe (Asurmendi et al., 2007). In brief, plant tissue was homogenized, and 400 μl of 0.5 M phosphate-extraction pH 7.0 buffer was added (4.1 g Na_2_HPO4, 2.5 g NaH_2_PO_4_, 100 μl β-mercaptoethanol per 100 ml). The samples were purified using equal volumes (400 μl) of chloroform and 1-butanol. Viral particles were precipitated using separate volumes of 50 μl of 40% polyethyleneglycol 6000 and a 10% NaCl solution. The pellet was resuspended in a 1:50 dilution of phosphate buffer that lacked β-mercaptoethanol, and viral concentrations were quantified. The viral titer was estimated by measuring OD260 using a spectrophotometer. The titer was expressed in microgram of ORMV per 10 mg fresh weight. Each measurement was done in triplicates.

## STATISTICAL TREATMENT OF THE DATA

In all cases, the average and standard errors were calculated. The statistical significance of the experiments was confirmed by performing either a Students *t*-test (two-tailed paired or non-paired) or a Single factor ANOVA. Statistical analyses were performed using the MS Excel software and Microcal Origin 6.0.

## Conflict of Interest Statement

The authors declare that the research was conducted in the absence of any commercial or financial relationships that could be construed as a potential conflict of interest.
